# The Role of the Right Hemisphere in Emotional and Behavioral Disorders of Patients With Frontotemporal Lobar Degeneration: An Updated Review

**DOI:** 10.3389/fnagi.2019.00055

**Published:** 2019-03-19

**Authors:** Guido Gainotti

**Affiliations:** ^1^Institute of Neurology of the IRCCS Fondazione Policlinico Gemelli, Catholic University of Rome, Rome, Italy; ^2^Department of Clinical and Behavioral Neurology, IRCCS Fondazione Santa Lucia, Rome, Italy

**Keywords:** laterality of emotions, the “right hemisphere” hypothesis, the “valence hypothesis”, asymmetrical forms of frontotemporal dementia, emotional and behavioral disorders, positive and negative emotions

## Abstract

**Background:** Two main models have been advanced to explain the asymmetries observed in the representation and processing of emotions. The first model, labeled “the right hemisphere hypothesis,” assumes a general dominance of the right hemisphere for all emotions, regardless of affective valence. The second model, named “the valence hypothesis,” assumes an opposite dominance of the left hemisphere for positive emotions and the right hemisphere for negative emotions. Patients with frontotemporal lobar degeneration (FTLD) could contribute to clarifying this issue, because disorders of emotional and social behavior are very common in FTLD and because atrophy, which affects the antero-ventral part of the frontal and temporal lobes, can be clearly asymmetric in the early stages of this disease.

**Objective:** The main scope of the present review therefore consists of evaluating if results of investigations conducted on emotional and behavioral disorders of patients with right and left FTLD, support the “right hemisphere” or the “valence” hypothesis.

**Method:** A thorough review of behavioral and emotional disorders in FTLD patients, found that 177 possible studies, but only 32 papers met the requested criteria for inclusion in our review.

**Results:** Almost all (25 out of 26) studies were relevant with respect to the “right hemisphere hypothesis” and supported the assumption of a general dominance of the right hemisphere for emotional functions, whereas only one of the six investigations were relevant with respect to the “valence hypothesis” and were in part consistent with this hypothesis, though these are also open to interpretation in terms of the “right hemisphere” hypothesis.

**Conclusions:** This study, therefore, clearly supports the model of a general dominance of the right hemisphere for all emotions, regardless of affective valence.

## Introduction

Even if most authors acknowledge that important asymmetries exist in the representation and processing of emotions, the nature of these asymmetries remains controversial. Two main models concerning the relationship between emotions and brain laterality have been advanced. The first model, labeled “the right hemisphere hypothesis” (Gainotti, 1972, 2012; Borod et al., 1998; Mandal and Ambady, 2004), posits a general dominance of the right hemisphere for all emotions, regardless of affective valence. This model was proposed by Gainotti (1972) on the basis of clinical observations made in patients with right and left hemisphere lesions, because (aphasic) left brain-damaged patients typically showed “catastrophic reactions” in their frustrating attempts of verbal expression, whereas patients with severe right hemisphere lesions typically showed an “indifference reaction” in their defects and disabilities. Since this “indifference reaction” included a denial or a lack of concern for the disability and for failures during the neuropsychological examination and a tendency for patients to joke in a fatuous, ironic or sarcastic manner, Gainotti (1972) proposed that this pattern of behavior was much more abnormal than the catastrophic reactions of left brain-damaged patients. It was considered that abnormal emotional reactions were due to the major involvement of the right hemisphere in emotional functions, in agreement with the relationship that exist between language disturbances of left brain-damaged patients and the dominance of the left hemisphere for language. In the following years, this interpretation, assuming a general dominance of the right hemisphere for emotional functions, was confirmed by experimental and clinical investigations which studied the non-verbal communicative aspects of emotions in normal subjects and in patients with unilateral brain lesions. Most of these studies showed, in fact, a general dominance of the right hemisphere, both in comprehension and in facial and vocal expression of positive and negative emotions (see Borod et al., 1998; Mandal and Ambady, 2004 and Gainotti, 2018 for reviews).

The second model, “the valence hypothesis” (Ahern and Schwartz, 1979; Reuter-Lorenz and Davidson, 1981; Rodway et al., 2003), assumes an opposite dominance of the left hemisphere for positive emotions and of the right hemisphere for negative emotions. This model is based on results obtained on a limited number of investigations, which studied the non-verbal communicative aspects of emotions in normal subjects and in patients with unilateral brain lesions. Thus, Reuter-Lorenz and Davidson (1981), Reuter-Lorenz et al. (1983), and Natale et al. (1983), studying emotional comprehension with a tachistoscopic lateralized presentation of emotional faces to the right and left visual field, found a right hemisphere advantage for negative emotions and a left hemisphere advantage for positive emotions. In a similar vein, Sackeim and Gur (1978) and Sackeim and Grega (1987), using composite photographs of posed positive or negative emotions (created using two left or two right half faces), found a left-sided facial asymmetry in the expression of negative but not of positive emotions (see Gainotti et al., 1993; Mandal and Ambady, 2004 and Killgore and Yurgelun-Todd, 2007 for detailed critical reviews).

A variant of this model, proposed by Davidson (1983, 1992) and labeled “the approach-withdrawal” hypothesis, maintains that brain asymmetries observed during positive and negative emotions are not related to the valence of the emotional stimuli, but to the motivational system engaged by those stimuli. According to this model, the left prefrontal cortex (PFC) might be involved in a system facilitating an approach to appetitive stimuli, whereas the right PFC might be involved in a system facilitating withdrawal from aversive stimuli. A different set of data has been used to support, on one hand, the “right hemisphere hypothesis” and the “valence hypothesis” and, on the other hand, the “approach-withdrawal” hypothesis. Data supporting the “right hemisphere hypothesis” and the “valence hypothesis,” respectively, have been obtained by studying the comprehension and expression of positive and negative emotions at the facial or vocal level in normal subjects and in patients with focal unilateral brain damage. On the contrary, most data supporting “the approach-withdrawal” hypothesis have been obtained by studying EEG asymmetries at the level of the frontal lobes during positive and negative emotions or situations involving approach or avoidance/withdrawal components. However, the use of frontal EEG asymmetries to assess emotion or motivation has recently been criticized on the basis of methodological and conceptual reasons. For instance, Miller et al. (2013) have noticed that treating the left- and right- frontal lobes as the units of analysis can be misleading because hemodynamic and electromagnetic neuroimaging studies suggest considerable functional differentiation in specialization and activation of subregions of the frontal cortex, including their connectivity to each other and to other regions. Important methodological objections have also been advanced by Allen et al. (2018) and Reznik and Allen (2018), who have shown that there is no sufficient evidence to support the relationship between frontal EEG asymmetries and emotional or motivational systems. In contemporary clinical science, studies aiming to check these models, with neurological patients, have become much less prevalent than investigations using functional imaging, but results of these studies have failed to clearly support both the “right hemisphere” and the “valence” hypothesis or the “approach-withdrawal” hypothesis (see Wager et al., 2003 and Fusar-Poli et al., 2009 for meta-analytic reviews). These rather disappointing results must, however, be considered with caution, because constraints and limits imposed by functional imaging methodologies constitute a far from ideal environment for studying full-blown emotional and social processes. Thus, Levenson et al. (2014) noticed that, even if it were possible to induce strong emotions in the scanner, huge signal artifacts would be produced by the associated muscular activity in the body and face. Therefore, most emotional and social research conducted in the scanner fall into the realm of social and emotional cognition, where the focus is on how we think and make judgments about social and emotional processes, rather than on real emotions as they unfold in real time. In patients with brain damage, on the contrary, different kinds of emotional behavior can be observed under controlled conditions in the laboratory or in more naturalistic conditions in the patient's natural environment. In recent years the interest of clinical researchers has shifted from patients with focal brain damage to patients with degenerative brain diseases, and in particular to patients with frontotemporal lobar degeneration (FTLD), because disorders of emotional and social behavior are very common in these patients and because atrophy, which affects the antero-ventral parts of the frontal and temporal lobes can be clearly asymmetric in the early stages of this disease. FTLD is the pathological substrate associate with Frontotemporal dementia (FTD), which is, indeed, a clinically heterogeneous disease in which both frontally predominant (fvFTD) and temporally predominant (tvFTD) subtypes have been described. Typically, tvFTD has been defined by the presence of deficits in language and semantic knowledge and is for this reason often labeled Semantic Dementia (SD). On the other hand, fvFTD has been defined by its behavioral features, and is for this reason often labeled “behavioral variant Frontotemporal Dementia” (bvFTD) even if many studies (e.g., Bozeat et al., 2000; Snowden et al., 2001; Rosen et al., 2002a; Liu et al., 2004; Rascovsky et al., 2011) have shown that both of these anatomo-clinical syndromes may be associated with behavioral disturbances. Furthermore, several authors (e.g., Snowden et al., 1996, 2004; Thompson et al., 2003; Seeley et al., 2005, 2008) have shown that in the early stages of FTD the atrophy affecting the frontal and (even more) the anterior temporal lobes (ATLs) can be clearly asymmetric. Since many other studies (e.g., Miller et al., 1993; Edwards-Lee et al., 1997; Mychack et al., 2001; Perry et al., 2001; Seeley et al., 2005; Irish et al., 2013; Binney et al., 2016) have shown that emotional and behavioral disorders may be on the foreground when atrophy prevails in the right frontal or temporal lobes, we believe that a careful review of these studies could help to clarify the nature of the relationships that exist between emotions and brain laterality. A further source of information on this subject was represented by investigations conducted in patients with FTLD, in which correlations had been found between specific emotional disorders and atrophy or dysfunction of equally specific lateralized cortical or subcortical structures. The main scope of the present review therefore consists of evaluating if the results of investigations conducted in patients with FTD, clearly support the “right hemisphere” or the “valence” hypothesis or if it cannot clarify the nature of asymmetries that exist in the representation and processing of emotions.

## Methods

In this review, to check the above mentioned models, we considered all studies found in the literature that investigated the influence of asymmetrical atrophies on emotional and behavioral disorders in patients with FTLD in the last 25 years (from 1993 to 2018). This time interval was chosen because, even if there had been many earlier papers evaluating the “right hemisphere” hypothesis in other neurological diseases (e.g., Coffey, 1987; Gainotti and Caltagirone, 1989; Borod et al., 1998), the first investigations dealing with the relationships between emotional/behavioral disorders and laterality of atrophy in FTLD were published 25 years ago (Miller et al., 1993). In the earliest studies authors focused on behavioral disorders and the assessment of emotional disturbances was based more on a clinical evaluation than on specific neuropsychological tests. On the contrary, in more recent investigation's specific emotional disorders were studied with specific sophisticated testing procedures. Since data from several studies suggested that strong interconnections exist between behavioral and emotional disorders, both kinds of studies were included in our review, even if a separate analysis of these data was also undertaken. With this aim in mind, we performed searches using PubMed and Medline for studies including diagnostic keywords (“degenerative diseases,” “semantic dementia,” “primary progressive aphasia,” and “FTD”) in conjunction with relevant content keywords related to “behavioral and emotional disorders,” “positive and negative emotions,” and “lesion laterality.” The initial search yielded 168 studies across the databases and 55 additional publications were identified from references of the obtained articles. After removal of 46 duplicates, papers to be included in the review were selected within the 177 remaining publications.

### Inclusion Criteria

The following criteria were used for the selection of papers to be included in the review: (a) the presence of data allowing the evaluation of the relationships between emotional and behavioral disorders and the prevalent lesion of the right or left frontal or temporal lobes; (b) the use of precise clinical definitions or neuropsychological measures of the emotional/behavioral disorders considered in the study, in conjunction with (c) a comparison of results obtained on these measures by patients showing a prevalence of left-sided or right-sided lesions (atrophies or FDG-PET hypometabolism) or (d) a study of the correlation between specific emotional disorders and atrophy or hypometabolism of equally specific left-sided or right-sided structures. Most studies screened according to the inclusion criteria were discarded because they had not considered laterality as an important variable (*N* = 96), whereas some were excluded because of insufficient diagnostic criteria (*n* = 27) or of methodological inconsistencies (*N* = 22). Only 32 studies met both the first two and the third or the fourth criterion and are reported in Table 1. Most of these investigations (*N* = 26) were mainly relevant with respect to the “right hemisphere hypothesis,” whereas few (*N* = 6) were considered as relevant with respect to the “valence hypothesis.” Some investigations that reported interesting observations, but met only in part the requested criteria, were not included in Table 1, but were considered in the Discussion section.

**Table 1:** Synthetical description of investigations which have studied emotional and behavioral disorders in patients with frontal or temporal variant of Fronto-Temporal Degeneration, evaluating a prevalence of left-sided or right-sided atrophy in patients with emotional disorders or a correlation between specific emotional disorders and atrophy or dysfunction of lateralized cortical or subcortical structures.

**Authors Methods and results**
Miller et al. (1993) Reported clinical, neuropsychological, and SPECT characteristics of five patients with progressive right frontotemporal degeneration (FTD). Flattened affect, compulsions and behavioral disinhibition of right FTD patients contrasted dramatically with normal behavior and emotional status of patients with left FTD. Edwards-Lee et al. (1997) Matched the clinical, neuropsychological, and neuropsychiatric features of five patients with predominantly left-sided and five with predominantly right-sided temporal variant of FTD (tvFTD). In left-sided patients', aphasia was usually the first and most severe clinical abnormality, whereas right-sided patients showed behavioral disorders characterized by irritability, impulsiveness, and fixed facial emotional expressions. Mychack et al. (2001) Tested the hypothesis that right-sided FTD may be associated with socially undesirable behavior, classifying on visual inspection of SPECT scans, 12 patients as having predominantly right-sided and 19 as having predominantly left-sided FTD. Eleven of 12 right-sided, but only two of 19 left-sided FTD patients had socially undesirable behavior as an early presenting symptom. The authors concluded that right-sided frontotemporal degeneration is associated with socially undesirable behavior. Perry et al. (2001) Assessed semantic memory, processing of emotional facial expression, and emotional prosody in two patients with right and left tvFTD. The subject with predominantly left sided atrophy had severe semantic impairment but normal performance on all emotional tasks. In contrast, the subject with right sided atrophy showed impaired recognition of emotion from faces and voices, loss of empathy and fixed facial expressions. Rosen et al. (2002b) Examined the comprehension of facial expressions of emotion in nine individuals with tvFTD, and correlated performance on this measure with atrophy of the amygdala, anterior temporal lobe (ATL), and orbitofrontal cortex (OFC). They also investigated the relationship between specific emotions and regional cerebral volumes. Emotional comprehension (evaluated with a composite measure of performance on sadness, anger, and fear) was correlated with atrophy in the right amygdala, whereas performance on both happiness and sadness measures was significantly correlated with right amygdala and right OFC volume. Liu et al. (2004) Compared behavioral dysfunction in anatomically defined temporal and frontal variants of frontotemporal dementia (tvFTD and fvFTD) and Alzheimer's disease. They showed that in FTD, the presence of behavioral disorders was associated with decreased volume in right-hemispheric regions. Rosen et al. (2005) Examined neuroanatomical correlates of behavioral abnormalities, as measured by the Neuropsychiatric Inventory, in 148 patients with dementia using voxel-based morphometry. Their data strongly supported the involvement of distinct regions on the medial wall of the right frontal lobe in regulating different social and emotional behaviors. Seeley et al. (2005) Investigated, in patients with left vs. right tvFTD, the first symptoms and the timing of subsequent symptomatology. In most patients with right > left temporal atrophy the first symptoms consisted of a behavioral syndrome, characterized by emotional distance, irritability, and disruption of physiologic drives, whereas in most patients with left temporal lobe variant the first symptoms consisted of anomia, word-finding difficulties, and repetitive speech. Rankin et al. (2006) Investigated the neuroanatomic basis of empathy in 123 patients with different degenerative diseases, correlating the Empathic Concern and Perspective taking scores with structural MRI brain volume using voxel-based morphometry (VBM). Voxels in the right temporal pole, the right fusiform gyrus, the right caudate and right subcallosal gyrus correlated significantly with total empathy score. Rosen et al. (2006) Examined, in 50 patients with neurodegenerative disease, the neuroanatomical correlates of impaired recognition of emotions (as measured by the Florida Affect Battery) using VBM. Consistent with previous studies, recognition accuracy in the group was poor for negative emotions (fear, anger, and sadness) and good for happiness. For negative emotions, accuracy was correlated with a region in the right lateral inferior temporal gyrus[Brodman's area (BA) 20] extending into the right middle temporal gyrus (BA 21). Viskontas et al. (2007) Reviewed the behavioral and neuropsychological changes that accompany OFC atrophy in the earliest stages of FTD. They argued that selectively vulnerable in FTD are phylogenetically new neurons found in this region, called von Economo neurons (VENs), which are 30% more abundant in the right than in left hemisphere and could play a role in the conscious experience of emotions. Werner et al. (2007) Assessed emotional reactivity and emotion recognition in 28 FTLD patients and 16 controls. For emotional reactivity, greater happy facial behavior during the happy film was associated with greater lobar volumes in the right temporal and right frontal lobes, whereas, greater sad facial behavior during the sad film was associated with greater neuronal volume in the right frontal lobe. For emotional recognition, lower volumes were associated with poorer emotion recognition for fear in the right temporal and for sadness in the left frontal lobes. Chan et al. (2009) Compared the pattern of atrophy and the clinical features of 20 patients with predominant right temporal lobe atrophy with those observed in a group of patients with semantic dementia and prevalent left-sided temporal lobe atrophy. A variety of behavioral disorders including social disinhibition, behavioral rigidity, and aggressive behavior was observed in both patient groups. However, all of these symptoms, with the exception of behavioral rigidity, were more frequently observed in in patients with right temporal lobe atrophy than in those with left-sided semantic dementia. Massimo et al. (2009) Investigated the neuroanatomical correlations of apathy and disinhibition (as measured by the Neuropsychiatric Inventory) in FTD patients. Apathy was correlated with bilateral medial, orbital, inferior and dorsolateral frontal areas and with right middle temporal and right caudate regions. Disinhibition was associated with bilateral orbital and inferior frontal, bilateral insula, and with right middle temporal regions. Kipps et al. (2009) Tested 26 individuals with bvFTD using The Awareness of Social Inference Test (TASIT) to assess their ability to identify emotion and sarcasm in video vignettes. A multivariate imaging analysis showed that the sarcasm (and emotion) recognition deficit was dependent on a circuit involving the lateral orbitofrontal cortex, insula, amygdala, and temporal pole, particularly on the right. Rankin et al. (2009) Investigated the neuroanatomy underlying failure to understand sarcasm from dynamic vocal and facial paralinguistic cues in 90 subjects with neurodegenerative disease. Poorer sarcasm comprehension was predicted by smaller volume in bilateral posterior parahippocampal (PHc), temporal poles, and R medial frontal pole. According to the authors, the right medial frontal pole could identify the social context as sarcastic, and recognize the speaker's paradoxical intentions. Rosen et al. (2010) Examined the neuroanatomical basis of self-appraisal (that gives us the ability to recognize our limits and choose our activities accordingly) in a mixed group of 39 individuals with neurodegenerative diseases, using VBM and an objective, neuropsychologically-based measure of self-appraisal accuracy. They showed that self-appraisal accuracy was correlated with tissue content in the right ventromedial prefrontal cortex. Eslinger et al. (2011) Administered the IRI, a standardized, 28-item inventory of empathy that yields a total score, as well as four subscale scores (Perspective-Taking, Fantasy, Empathic Concern, and Personal Distress), to 26 patients with bvFTD and 16 normal controls. Voxel-based morphometry revealed that reduced empathic perspective-taking was correlated with widespread areas of the right dorsolateral prefrontal cortex, extending to left superior mesial premotor cortices, right parietal regions, and left superior temporal gyrus, whereas empathic emotions were related to right medial frontal atrophy. Goodkind et al. (2012) Examined the neural correlates of continuously tracking dynamically changing emotions, asking 59 patients with diverse neurodegenerative diseases to track continuously how positive or how negative felt the character in a film clip. Low tracking accuracy was primarily associated with gray matter loss in the right lateral orbitofrontal cortex (OFC). 20. Couto et al. (2013) Studied the neuroanatomic correlates of emotional and social (ToM) deficits in bvFTD and progressive non-fluent aphasia (PNFA) patients. Emotion deficits were associated to atrophy of FIC in bvFTD and of right temporal pole plus bilateral insula in PNFA. TcM defects correlated with FIC in bvFTD and with right insula and temporal pole in PNFA. Irish et al. (2013) Administered tests of emotion processing and interpersonal functioning to 10 right and 10 left SD/tvFTD patients. Left SD cases displayed marked difficulties in the recognition of all negative emotions and surprise on the Ekman 60 faces test, whereas right SD patients showed profound deficits in the recognition of both positive and negative facial emotions, scoring significantly poorer than the left SD group for the recognition of anger and happiness. Sturm et al. (2013) Investigated the neural correlates of self-conscious emotional reactivity in 27 patients with bvFTD and in 33 healthy older controls, who participated in an embarrassing karaoke task in which they watched a video clip of themselves singing. Right pregenual anterior cingulate cortex (pACC) gray matter volume was the only brain region that was a significant predictor of self-conscious emotion. Irish et al. (2014) Assessed with a simple cartoons task “theory of mind” impairments in patients with left predominant semantic dementia (SD/tvFTD), behavioral-variant FTD and Alzheimer's disease. VBM analyses revealed that atrophy in right ATL structures, correlated significantly with theory of mind impairments in the left SD group, suggesting that in this disease the onset of social cognitive deficits is heralded by the progressive development of pathology into the contralateral right hemisphere. Cerami et al. (2015) Administered to 17 probable mild bvFTD patient's social cognition tasks assessing the recognition of basic emotions and the attribution of emotions and intentions and correlated these scores with regional FDG-PET measures. FDG-PET hypometabolism was highly correlated to the emotional recognition and attribution performances in the right amygdala, R temporal pole, and R middle cingulate cortex. The damage of the right amygdala was the main responsible of emotion recognition and attribution deficits. Downey et al. (2015) Assessed social cognition with an abbreviated TASIT comprising emotion identification and sarcasm identification subtests in a cohort of 29 bvFTD patients and of 15 SD/tvFTD patients. Identification of canonical emotions and sarcasm deficits were correlated with white matter tract alterations particularly affecting frontotemporal connections in the right cerebral hemisphere. Sturm et al. (2015) Measured happiness reactivity in 96 patients with FTD who watched two film clips designed to elicit respectively happiness and sadness. Whole-brain VBM analyses revealed that atrophy in predominantly left hemisphere fronto-striatal emotion regulation systems was associated with greater happiness facial behavior during the film. 27. Binney et al. (2016) Compared results obtained by 21 left and 12 right patients with Primary Progressive Aphasia (PPA) on tasks of exception word reading, familiarity judgments on famous faces, and affect processing. The ability to recognize facial expression of emotions was assessed using the Affect Matching subtest of the Comprehensive Affect Testing System. Right greater than left ATL atrophy was associated primarily with early changes in personality and behavioral disturbances such as decreased empathy, blunted affect, and deficits in receptive emotional processing. Dermody et al. (2016) Studied the neural bases of cognitive and affective empathy deficits using the Interpersonal Reactivity Index (IRI) in 25 AD and 24 bvFTD patients. Cognitive empathy was comparably compromised in AD and bvFTD, whereas affective empathy was impaired exclusively in bvFTD. Reduced empathic concern in bvFTD was associated with atrophy in the left orbitofrontal, inferior frontal, and insular cortices, and the bilateral mid-cingulate gyrus. Kumfor et al. (2016) Asked to 22 predominantly left and 9 predominantly right ATL atrophy to complete a Face and Emotion Processing Battery and the Cambridge Behavioral Inventory. Correlational analyses revealed that emotion recognition was associated with right temporal pole, right medial orbitofrontal, and right fusiform integrity, while changes in motivation were associated with right temporal pole cortical thinning. Multani et al. (2017) Compared emotion detection, using the emotion evaluation task (EET) of the TASIT, across the three PPA variants and healthy controls (HC), and related them to white matter tract integrity and cortical degeneration. Performance on EET was related to the right uncinate fasciculus (UF), superior longitudinal fasciculus (SLF), and inferior longitudinal fasciculus (ILF) integrity. Regression analysis revealed that EET performance primarily relates to the right UF integrity. Perry et al. (2017) Administered a series of tasks involving pleasant, unpleasant, and neutral olfactory stimuli, designed to separate distinct phases of reward processing, in 25 patients bvFTD and 21 control subjects. Voxel-based morphometry of patients with bvFTD revealed that the inability to subjectively differentiate the valence of pleasant and unpleasant odors correlated with atrophy in right ventral mid-insula and right amygdala. Sturm et al. (2017) Developed a novel task (the “Giving Game”) to measure prosocial behavior. On each trial of the Giving Game, participants (20 bvFTD, 15 AD, and 39 HC) decided how much money to offer to the experimenter, and prosocial giving was the total amount that they gave to the experimenter when it cost them nothing to give. Voxel-based morphometry showed that lower prosocial giving was associated with atrophy in the right pulvinar nucleus of the thalamus, whereas greater prosocial giving was associated with atrophy in the left ventral striatum.

## Results

A short descriptive survey of the 32 studies included in our review is reported in Table 1.

Descriptive data reported in Table 1 provide an idea of the complexity and heterogeneity of the research lines considered in our review. These investigations, in fact, though all relevant in relation to the subject of our review, investigated a large array of clinical syndromes as well as behavioral disorders (e.g., papers 1–3, 6–8, 11, and 13–14), resulting (at least in part) from emotional disturbances or emotional disorders *per se*. Furthermore, even true emotional disorders involved two different categories of emotions. In some cases, basic emotions were considered (e.g., papers 5, 10, 12, 21, 24, 27, and 29), which are regarded as innate and provide high survival values (Ekman, 1984, 1992). In other cases, they considered “complex,” “social,” and “self-conscious emotions” (e.g., 9, 15–18, 22–25, 28, and 30–31), which are subtended by social norms (Ekman, 1984, 1992) and require an appreciation of the self in a social context (Tangney, 1999).

A more analytical tabulation of patients (number of participants), emotional and behavioral disorders (clinical diagnosis, tests undertaken and neuroanatomical assessment) and study methodology (group comparison or correlational analysis) of papers: (a) dealing with behavioral disorders; (b) relevant with respect to the “right hemisphere hypothesis” and (c) specifically concerning the “valence hypothesis” is therefore, reported in Table 2.

**Table 2:** More detailed account of papers included in the present review and: (a) dealing with behavioral disorders; (b) relevant to the “right hemisphere hypothesis” and (c) specifically concerning the “valence hypothesis.”

**Authors**	**Patients**	**Emotional and behavioral disorders**		**Study methodology**	**Results**
		**Investigation of target disorders**	**Assessment of location and laterality of lesions**		**Side of involvement**
**PAPER DEALING WITH BEHAVIORAL DISORDERS**					
1. Miller et al. (1993)	5 Right vs. Left FTD patients	Evaluation of behavioral disorders on medical records	Asymmetrical perfusion on SPECT	Group comparison. Clinical, SPECT and neuropsychological data	Greater on the Right. Flattened affect, compulsions and behavioral disinhibition prevailed in right FTD patients
2. Edwards-Lee et al. (1997)	5 patients with Right vs. 5 with Left FTD	Evaluation of behavioral disorders on medical records	Asymmetrical perfusion on SPECT	Group comparison Clinical, SPECT and neuropsychiatric features	Greater on the Right. Irritability, impulsiveness and decreased facial expressions prevailed in right FTD patients.
3. Mychack et al. (2001)	12 patients with Right vs. 19 with Left FTD	Blind evaluation of a list of socially abnormal behaviors	Lesion laterality was assessed on visual inspection of SPECT scans	Correlational analysis. Association between right frontotemporal atrophy and social behavior	Greater on the Right (11/12 right- and 2/19 left-sided FTD patients had socially abnormal behaviors as a presenting symptom)
6. Liu et al. (2004)	51 patients with FTD, 22 with AD and 20 Normal Controls	Behavioral abnormalities as measured by the Neuropsychiatric Inventory (NPI)	Relationship between behavioral disorders and volumetric measurements of frontal, ATL and VMFC structures	Correlational analysis. Volumetric measurements of right and left frontal structures, ATL and Amygdala	Greater on the Right. A factor loaded in on disinhibition and elation was associated with decreased volume in the right ATL and right and left VmFC regions.
7. Rosen et al. (2005)	148 demented patients (52 AD, 39 FTD, 23 Sem. Dem.)	Behavioral abnormalities, as measured by the Neuropsychiatric Inventory	The anatomical correlates of behavioral abnormalities were studied in dementia pts using VBM	Correlational analysis The anatomical correlates of behavioral abnormalities were studied using voxel-based morphometry	Greater on the Right. Behavioral dysfunction was correlated with tissue loss in several cortical regions in the right hemisphere
8. Seeley et al. (2005)	26 patients with tvFTD were identified and 6 patients with right tvFTD were matched with 6 with left tvFTD.	Clinical records were reviewed to identify the first symptoms	MRI volumes for the left and right ATL, amygdala, VmFC, and total frontal cortex (TFC) were computed.	Group comparison. The first symptom was behavioral in 4/6 right vs. 1/6 left tvFTD.	Greater on the Right. Emotional distance, irritability, and disruption of physiologic drives characterized the early behavioral syndrome
11. Viskontas et al. (2007)	Showed that OFC and insula are the most vulnerable regions in 12 FTD patients showing defects of emotion recognition and empathy	A link between fronto-insular von Economo neurons(VEN) and social behavior was suggested	Argued that these phylogenetically new neurons are involved in emotional functions	Review of the behavioral and neuropsychological changes that accompany OFC atrophy in FTD	Greater on the Right. von Economo neurons (VENs), which are 30% more abundant in the right than in left hemisphere are selectively vulnerable in FTD
13. Chan et al. (2009)	20 patients with right temporal lobe atrophy were compared with 10 Normal Controls and 10 left tvFTD patients	Clinical and volumetric MRI data were acquired from the London Dementia Research Centre (DRC)	Profiles of cognitive, behavioral and personality changes were obtained on each patient.	Group comparison. TvFTD patients exhibited a variety of behavioral disorders (mainly disinhibition, depression and aggressive behavior).	Greater on the Right. All behavioral symptoms, with the exception of behavioral rigidity, were more frequently observed in patients with right temporal lobe atrophy
14. Massimo et al. (2009)	Forty caregivers of patients with the clinical diagnosis of FTLD completed the NPI	Twelve neuropsychiatric symptoms and the associated caregiver distress were assessed	Optimized voxel-based morphometry identified significant atrophy in subgroups of FTLD patients	Correlational analysis. The greatest stressors for caregivers were apathy and disinhibition	Greater on the Right. Both apathy and disinhibition were correlated with bilateral frontal and right temporal areas
**PAPERS RELEVANT WITH RESPECT TO THE “RIGHT HEMISPHERE HYPOTHESIS”**					
4. Perry et al. (2001)	Assessed in four patients with right and left tvFTD various aspects of emotional comprehension and expression	Two patients were right-handed and two left-handed. Semantic memory, and emotional disorders were studied in each of them.	Empathy, emotional facial comprehension and expression and emotional prosody were investigated	Right / Left comparisons. Subjects with left sided atrophy had semantic impairment but normal performance on all emotional tasks.	Greater on the Right. Subjects with right sided atrophy showed impaired recognition of emotion from faces and voices, loss of empathy and fixed facial expressions.
9. Rankin et al. (2006)	Investigated the neuroanatomic basis of empathy in 123 neurodegenerative disease patients (30 fvFTD, 26 Sem. Dem., 38 AD) and 20 healthy controls.	The Interpersonal Reactivity Index (IRI) Empathic Concern and the Perspective taking scores were used to assess empathy	The neuroanatomy of empathy was studied with structural MRI brain volume using VBM	Correlational analysis. Voxels in the right temporal pole, the right fusiform gyrus, the right caudate and right subcallosal gyrus correlated significantly with total empathy score.	Greater on the Right. Results suggest that the right anterior temporal and medial frontal regions are essential for real-life empathic behavior.
15. Kipps et al. (2009)	Tried to quantify aspects of the behavioral disorder in 26 pts with bvFTD, 9 with AD and 16 healthy controls	The Awareness of Social Inference Test (TASIT) was used to assess their ability to identify emotion and sarcasm in video vignettes	Defect in recognition of sarcasm and other emotions was correlated to the underlying level of atrophy in socially relevant brain regions	Correlational analysis. The capacity of bvFTD patients to interpret negative emotion was a major factor underlying the deficit in sarcasm recognition.	Greater on the Right. The sarcasm (and emotion recognition) deficit was dependent on a circuit involving the lateral orbitofrontal cortex, insula, amygdala and temporal pole, particularly on the right
16. Rankin et al. (2009)	Failure to detect sarcasm was studied in 90 subjects (20 fvFTD, 11 Sem. Dem. 27 AD and 13 healthy controls)	The Social Inference—Minimal subtest of The TASIT was used to study sarcasm comprehension	Patients failing to comprehend sarcasm (SSR) performed poorer on emotion recognition tasks than patients who comprehended sarcasm	Correlational analysis. Small volume in bilateral posterior parahippocampal temporal poles, and R medial frontal pole predicted poor sarcasm comprehension	Greater on the Right. The temporal poles and, the right medial frontal pole could identify the social context as sarcastic, and recognize the speaker's paradoxical intentions
17. Rosen et al. (2010)	Examined the neuroanatomical basis of self-appraisal in 39 neurodegenerative disease patients	After having completed some cognitive tasks, patients evaluated if they thought having been average, above average, or below average.	An objective, measure of self-appraisal accuracy was correlated with VBM tissue content in the ventromedial prefrontal cortex (vmPFC).	Correlational analysis. Reduced right vmPFC volume was associated with greater overestimation of cognitive performance	Greater on the Right. Only the right vmPFC surpassed the statistical threshold in the analysis, of the data. However, at a less stringent statistical threshold, a possible relationship for left sided structures could also be suggested
18. Eslinger et al. (2011)	Apathy was studied in 26 patients diagnosed as bvFTD (*N* = 12), SD (*N* = 7 and) PNFA (*N* = 7) and their caregivers were studied. 16 NC were also studied.	Results obtained by patients at the IRI, a standardized, 28-item inventory of empathy, were correlated with structural MRI images using VBM	In addition to a total score the IRI yields four subscale scores (Perspective-Taking, Fantasy, Empathic Concern, and Personal Distress)	Correlational analysis. Reduced perspective-taking was related to atrophy of the right dorsolateral prefrontal cortex, extending to the left temporal areas. Empathic concern was related to right medial frontal atrophy.	Greater on the Right. Differences were found between anatomical correlates of (cognitive) empathic perspective-taking and (emotional) empathic concern, although both showed strong frontal-lobe correlations.
19. Goodkind et al. (2012)	Examined the neural correlates of continuously tracking changing emotions, asking 59 patients with neurodegenerative diseases (18 FTD, 13 SD, 15 AD) and 10 NCs to track continuously how positive or negative the character in a film clip felt	Tracking accuracy was assessed using videotaped vignettes of everyday social interactions that incorporate audio and contextual cues and identify the primary emotion expressed by the target	The relationship between tracking accuracy and regional brain tissue content was examined using voxel-based morphometry	Correlational analysis. Lower performance on the emotion tracking task was significantly correlated with gray matter tissue loss in: (a) a large region of voxels in the right orbitofrontal cortex and (b) smaller clusters of voxels in the triangularis portion of the right inferior frontal gyrus	Greater on the Right. Finding that the right OFC is critical to the ability to track dynamically-changing emotions is consistent with previous research showing right OFC involvement in both socioemotional understanding and modifying responding in changing situations
21. Couto et al. (2013)	Studied emotional and social deficits in12 bvFTD and 10 progressive non-fluent aphasia (PNFA) patients.	A test of facial emotion recognition and the Reading the mind in the eyes test (RMET) of ToM emotional inference were used	The patterns of atrophy related to emotional and social deficit were separately assessed in bvFTD and PNFA patients	Correlational analysis. In bvFTD patients both emotion deficits and ToM impairments were associated to atrophy of Fronto-Insular cortices (FIC). in PNFA emotion correlated with right temporal pole plus bilateral insula and ToM with right insula and temporal pole.	Slightly greater on the Right. Even if in bvFTD patients ToM and emotional defects were related to bilateral FIC lesions, in PNFA patients, emotion deficits and ToM impairments correlated more with right than with left insula and temporal pole.
22. Sturm et al. (2013)	Investigated the neural correlates of self-conscious emotional reactivity in 27 patients with bvFTD and in 33 healthy controls	Subjects participated in an embarrassing karaoke task in which they watched a video clip of themselves singing	Right pregenual anterior cingulate cortex (pACC) gray matter volume was a significant predictor of self-conscious emotion.	Correlational analysis. Smaller pACC volume was associated with attenuated physiological and behavioral self-conscious emotional reactivity	Greater on the Right. The right pACC plays a significant role that in the visceromotor responding that accompanies self-conscious emotion
23. Irish et al. (2014)	Determined the neural correlates of theory of mind performance in 11 patients with left SD, 10 with fvFTD, 10 AD and 14 NCs	A cartoon task that dissociates between physical and ToM understanding of humorous scenarios was used to assess theory of mind	Correlations between performance on the theory of mind task and regions of gray matter atrophy were investigated in each FTD patient group, combined with control subjects	Correlational analysis. ToM were related to gray matter intensity decrease in the right temporal fusiform cortex, and right inferior temporal gyrus, as well as the temporal poles and amygdala bilaterally.	Greater on the Right. Even if the left hemisphere was mainly affected in these patients, atrophy in right ATL structures, correlated significantly with theory of mind impairments
24. Cerami et al. (2015)	Administered to 17 mild bvFTD patient's social cognition tasks assessing the recognition of basic emotions and the attribution of emotions and intentions. Caregivers were given the IRI task to investigate patients' empathic attitude.	The Ekman 60-Faces test- and the Story-based Empathy task, assessing the recognition of basic emotions and the attribution of emotions and intentions were used	FDG-PET was analyzed using an optimized voxel-based SPM method at the single-subject and group levels.	Correlational analysis. At the group level, metabolic dysfunction in the right amygdala, temporal pole, and middle cingulate cortex was highly correlated to the emotional recognition and attribution performances.	Greater on the Right. The damage of the right amygdala was the main responsible of emotion recognition and attribution deficits
25. Downey et al. (2015)	Assessed social cognition in a cohort of 29 bvFTD patients, of 15 SD/tvFTD patients and of 37 normal controls.	An abbreviated TASIT comprising emotion identification and sarcasm identification subtests was used	Diffusion tensor imaging (DTI) was used to derive white matter tract correlates of social cognition performance and compared with the distribution of gray matter atrophy on voxel-based morphometry.	Correlational analysis. DTI associations of impaired social cognition were more consistent than corresponding gray matter associations.	Greater on the Right. Deficits in identification of canonical emotions and sarcasm, were correlated with white matter tract alterations particularly affecting frontotemporal connections in the right cerebral hemisphere.
27. Binney et al. (2016)	Compared results obtained by 21 left and 12 right patients with Semantic Dementia (svPPA) on tasks of reading, face recognition and affect processing	Recognition of facial expression of emotions was assessed using the Affect Matching subtest of the Comprehensive Affect Testing System (CATS)	Gray matter volumes of right and left temporal lobe sub-regions allowed to assess the neural substrates of naming, reading face processing and emotional disorders	Group comparison. Naming deficits and surface dyslexia were more severe in the left-group, whereas face familiarity judgments and affect processing were more impaired in the right- group.	Greater on the Right. Right greater than left ATL atrophy was associated primarily with early changes in personality and behavioral disturbances such as decreased empathy, blunted affect and deficits in receptive emotional processing
28. Dermody et al. (2016)	Studied the neural bases of cognitive and affective empathy deficits in 25 AD and 24 bvFTD patients and 22 NCs	Used the IRI to assess cognitive and affective empathy of apathy deficits	VBM was used to evaluate the neural bases of cognitive and affective apathy deficits	Correlational analyses. Reduced empathic concern in bvFTD was associated with atrophy in the left orbitofrontal and insular cortices, and the bilateral mid-cingulate gyrus	Greater on the Left. Loss of empathy in bvFTD reflects the deterioration of a distributed network of fronto-insular and temporal structures
29. Kumfor et al. (2016)	Made a longitudinal psychological and MRI study of 22 left and 9 right SD patients. Thirty-three AD patients and 25 healthy controls were included for comparison.	Patients completed the Cambridge Behavioral Inventory. and a Face Emotion Processing Battery (Face Perception and Matching, Emotion perception and Selection).	Weighted correlation coefficients were calculated for each individual between cortical thinning and behavioral performance.	Correlational analyses revealed that emotion recognition was associated with right temporal pole, right medial orbitofrontal and right fusiform integrity, while changes in motivation were associated with right temporal pole cortical thinning	Greater on the Right. Even in patients with an initial language presentation emotional changes reflect right anterior temporal and orbitofrontal cortex degeneration, underscoring the role of these regions in social cognition and behavior.
30. Multani et al. (2017)	Thirty-three patients with svPPA (*N* = 12), nfvPPA (*N* = 10), lvPPA(*N* = 9, and 32 HC underwent neuropsychological assessment, emotion evaluation task (EET), and MRI scan.	The EET is a subtest of the TASIT and consists of short video vignettes aiming to evaluate recognition of basic emotional expressions	Emotion detection was compared across the three PPA variants and healthy controls (HC), and related to white matter tract integrity and cortical degeneration	Correlational analysis. Performance on EET was related to the right uncinate fasciculus (UF), superior longitudinal fasciculus (SLF), and inferior longitudinal fasciculus (ILF) integrity.	Greater on the Right. Overall, the white matter tracts contributed more to accurate emotion recognition than the atrophied gray matter regions surrounding those tracts. Regression analysis showed that EET performance primarily relates to the right UF integrity.
32. Sturm et al. (2017)	Administered the “Giving Game,” (a new measure of prosocial behavior), to 74 participants (20 bvFTD, 15 AD, and 39 healthy controls	In this test, participants decided how much money to offer to the experimenter, Prosocial giving was the total amount that participants gave to the experimenter	Prosocial giving was lower in bvFTD than in healthy controls. Voxel-based morphometry was used to identify brain regions that were associated with prosocial giving.	Correlational analysis. Lower prosocial giving was associated with atrophy in the right pulvinar nucleus of the thalamus, whereas greater prosocial giving was associated with atrophy in the left ventral striatum	Greater on the Right. Simple acts of generosity deteriorate in bvFTD due to lateralized atrophy in reward-relevant neural systems that promote shared feelings of positive affect
**PAPERS SPECIFICALLY CONCERNING THE “VALENCE HYPOTHESIS”**					
5. Rosen et al. (2002b)	Used the Florida Affect Battery to examine the comprehension of facial emotional expressions in 9 patients with tvFTD, and 10 control subjects	Emotional comprehension was correlated with atrophy (as measured from MRI scans by region of interest analysis) in the amygdala, ATC and OFC.	In each of the facial affect subtests, photographs of faces depicting happiness, sadness, anger, fear or no emotion are presented.	Correlational analysis. Emotional comprehension (evaluated with a composite measure of performance on sadness, anger and fear) was correlated with atrophy in the right amygdala	Performance on both happiness and sadness measures was significantly correlated with right amygdala and right OFC volume.
10. Rosen et al. (2006)	Used the (FAB) to examine the comprehension of facial emotional expressions in 50 patients with neurodegenerative disease(25 FTD, 15 AD) and 5 healthy controls	Recognition accuracy in the group was poor for negative emotions (fear, anger and sadness) and good for happiness	Emotional comprehension was correlated with regional changes in gray matter tissue content using VBM	Correlational analysis across negative emotions (fear, anger and sadness), accuracy was correlated with a region in the right lateral inferior temporal gyrus	Emotion-specific effects For sadness, a region in the right superior temporal gyrus showed a significant correlation with performance independent of the effects for other emotions.
12. Werner et al. (2007)	Assessed in 28 FTD patients and 16 NCs emotional reactivity and emotional comprehension to emotional films	Film stimuli aimed to elicit fear, happy, and sad emotions. Emotional reactivity components were experience, facial expression and autonomic response.	The neural correlates of emotional reactivity and emotion recognition were investigated	Correlational analysis. For emotional comprehension greater volumes were associated with poorer emotion recognition for fear in the right temporal and for sadness in the left frontal and bilateral temporal cortices	For emotional reactivity, both greater happy facial behavior during the happy and sad facial behavior during the sad film were associated with greater lobar volume in the right frontal lobe
21. Irish et al. (2013)	Assessed 10 cases of predominantly right and 12 of left SD and 20 matched healthy controls on tests of emotion processing and interpersonal functioning.	The Ekman 60 and the TASIT were used to assess emotion processing, whereas the IRI was used as an index of the patient's present interpersonal functioning.	Recognition of 60 facial expressions across six basic emotions (anger, disgust, fear, happiness, sadness, surprise) was assessed using pictures and short video clips	Group comparison. On the Eckman 60 both SD subgroups were significantly impaired in the recognition of negative emotions, but right SD cases also showed disproportionate deficits in their capacity for empathic concern	Recognition of happiness was intact in left SD patients, whereas right SD patients displayed profound deficits in the recognition of all basic facial emotions, including the recognition of happiness.
26. Sturm et al. (2015)	Measured happiness reactivity in 96 patients with FTD (47 bvFTD, 33 tvFTD) and 34 healthy controls	Participants watched two film clips designed to elicit happiness and sadness while their facial behavior, physiological reactivity, and self-reported emotional experience were monitored.	Whole-brain VBM analyses was conducted in the patients to correlate emotional behavior with combined gray/white matter structural maps,	Correlational analysis revealed that atrophy in predominantly left hemisphere fronto-striatal emotion regulation systems was associated with greater happiness facial behavior during the happy film	Volume loss in predominantly left hemisphere regions was associated with greater happiness behavior and greater cardiovascular reactivity during the happy film in
31. Perry et al. (2017)	administered to 25 patients with bvFTD and 21 control subject a series of tasks involving pleasant, unpleasant, and neutral olfactory stimuli	Separate tasks assessed reward consumption and anticipation and effort to obtain reward. Subjective pleasantness ratings and skin conductance responses were evaluated	The underlying anatomy of the reward deficit in bvFTD and of the inability to differentiate pleasant from unpleasant stimuli was assessed with voxel-based morphometry	Correlational analysis. VBM of bvFTD patients revealed that the inability to subjectively differentiate the valence of pleasant and unpleasant odors correlated with atrophy in right ventral mid-insula and right amygdala.	Left dorsal anterior insula and frontal pole atrophy correlated with overly positive rating of unpleasant odors.

*AD, Alzheimer's Disease; ATL, Anterior temporal lobe; FTD, Fronto-temporal lobar degeneration (bv, behavioral variant; fv, frontal variant; sv, semantic variant; tv, temporal variant); NCs, Normal controls; OFC, Orbito-frontal cortex; PPA, Primary progressive aphasia (fl, fluent; lp, logopenic; nf, non-fluent); SD, Semantic dementia; VBM, voxel-based morphometry; VmFC, Ventro-medial frontal cortices*.

Results obtained in most (*N* = 26) of these investigations were mainly relevant to the “right hemisphere hypothesis” because they: (1) made a comparison between the prevalence of left-sided or right-sided lesions in patients with emotional or behavioral disorders or (2) showed a significant correlation between emotional disorders and atrophy or hypometabolism of left-sided or right-sided brain structures. Nine of these investigations, namely studies (1), (2), (3), (6), (7), (8), (10), (13), and (14) focused on behavioral disorders, whereas 17 investigations, namely studies (4), (9), (15), (16), (17), (18), (19), (20), (22), (23), (24), (25), (27), (28), (29), (30), and (32) focused on true emotional disorders and were relevant to the “right hemisphere hypothesis.” On the other hand, results obtained in a few (*N* = 6) of these investigations, namely studies (5), (10), (12), (21), (26), and (31) were relevant to the “valence hypothesis.” They made, indeed, a separate analysis of comprehension or production of positive and negative emotions and compared results obtained on these tasks by FTD patients, with a prevalence of left or right lesions (study 21) or correlated these patterns of emotional disorders with specific left-sided or right-sided structures (studies 5, 10, 12, 26, and 31).

### Results of Investigations Mainly Dealing With Behavioral Disorders

Data reported in Table 2 show that all investigations dealing with behavioral disorders (at least in part resulting from emotional disturbances) were consistent with the assumption of a general dominance of the right hemisphere for emotional functions. In fact, all these investigations demonstrated a prevalence of behavioral disorders in FTD patients with atrophy or hypometabolism of right-sided neural structures (studies 1, 2, 3, 8, and 13) or a significant correlation between behavioral disorders and atrophy or hypometabolism of right fronto-temporal structures (studies 6, 7, and 14). This was true irrespective of the nature of the reported behavioral disorder (which consisted of disinhibition in studies 1, 2, 6, 8, 13, and 16, of apathy in studies 1, 13, and 14 and of aggressive behavior in studies 2, 8, and 13) and of the disease stage that was considered. This behavioral disorder was, in fact, an observed symptom in papers 3 and 8, whereas it was observed in the moderate-to-advanced stages of the disease in the other papers.

### Results of Investigations Relevant With Respect to the “Right Hemisphere Hypothesis”

As in the case of investigations that mainly dealt with behavioral disorders, the results of studies on emotional disorders and those which were considered as relevant to the “right hemisphere hypothesis,” supported the assumption of a general dominance of the right hemisphere for emotional functions. Even in this case, in fact, almost all (i.e., 16 out of 17) investigations demonstrated either a greater emotional impairment in FTD patients, showing a prevalent atrophy of right-sided brain structures (studies 4 and 27) or a significant correlation between emotional disorders and atrophy, or hypometabolism of the right fronto-temporal structures (studies 9, 15, 16, 17, 18, 19, 21, 22, 23, 24, 25, 29, 30, and 32). In correlational study 28 (Dermody et al., 2016) only, the emotional defect (reduced empathic concern) was associated with prevalent atrophy in left-sided structures (the left orbitofrontal and insular cortices) in bvFTD patients.

Therefore, irrespective of the methodology used (group comparison vs. correlational analysis), results of investigations aiming to check the “right hemisphere hypothesis,” strongly supported the assumption of a general dominance of the right hemisphere for emotional functions. Obviously, this does not mean that the left fronto-temporal structures do not play a relevant role in emotional functions. Even when putting aside results obtained by Dermody et al. (2016), in all the other correlational studies, emotional disorders were associated to atrophy of both the right- and left- anterior temporal, insular and orbitofrontal cortices, although the correlations were stronger in right- than in left-sided structures.

### Results of Investigations Specifically Concerning the “Valence Hypothesis”

In the study (5), Rosen et al. (2002b) examined the comprehension of facial expressions of emotion in nine individuals with tvFTD and showed that performance on recognition of facial expressions (evaluated with a composite measure of performance on sadness, anger and fear) correlated with atrophy in the right amygdala. The same authors also investigated the relationship between comprehension of specific emotions and regional cerebral volumes and showed that both happiness and sadness measures were significantly correlated with the right amygdala and the right orbito-frontal cortex (OFC) volume. Thus, the correlation between a composite measure of performance on negative emotions (sadness, anger, and fear) and atrophy in the right amygdala, could be consistent with the “valence hypothesis,” but the fact that both happiness and sadness measures were significantly correlated with the right amygdala and OFC volumes, contradicts this model. Equally inconclusive results were obtained in study (10) by Rosen et al. (2006) and in study (12) by Werner et al. (2007). The first study used the Florida Affect Battery (Bowers et al., 1989) to assess recognition of emotions in 50 patients with neurodegenerative disease and correlated performance on recognition of facial expressions with regional changes in gray matter tissue content, using voxel-based morphometry. The second study compared emotional reactivity and emotional comprehension of emotional films designed to elicit fear, happy, and sad emotions in FTD patients and healthy controls. Consistent with previous studies, Rosen et al. (2006) found that the recognition accuracy in neurodegenerative patients was poor for negative emotions (fear, anger, and sadness) and good for happiness. They also found, however, that tissue content in a region in the right lateral inferior temporal gyrus [Brodman's area (BA) 20] extending into the right middle temporal gyrus (BA 21), was correlated with the accuracy of recognition of negative emotions. On the other hand, Werner et al. (2007) reported two data consistent with the “right hemisphere hypothesis.” The first was that the generation of both happy facial expressions to happy films and sad facial expressions to sad films, were associated with greater lobar volume in the right frontal lobe. The second was that regional brain atrophy was associated with poorer emotion recognition for fear in the right temporal and for sadness in the left frontal and bilateral temporal cortices. The fact that accuracy in recognition of negative emotions was positively correlated with tissue content of the right temporal structures, by Rosen et al. (2006), and with left frontal atrophy by Werner et al. (2007) could in part support the “valence hypothesis.” However, inconsistent with this interpretation are results obtained in study (21) by Irish et al. (2013), in study (26) by Sturm et al. (2015) and in study (31) by Perry et al. (2017), that studied positive and negative emotions in different subgroups of FTD patients, using different experimental paradigms. Irish et al. (2013) compared results, obtained by two groups of predominantly right (*n* = 10) and left (*n* = 12) SD patients, on the Ekman 60-Faces test of emotion processing (Young et al., 2002). In this test, left SD cases displayed marked difficulties only in the recognition of negative emotions, whereas recognition of happiness was intact. In the same task, right SD patients displayed profound deficits in the recognition of all basic facial emotions, including the recognition of happiness, and scored significantly worse than the left SD patients in the recognition of anger and happiness. These results, which showed that right SD patients scored worse than the left SD patients in the recognition of happiness, were clearly inconsistent with the “valence hypothesis,” because, according to this model, recognition of happiness should be subtended by the left hemisphere. Equally, at variance with the “valence hypothesis” were data obtained by Sturm et al. (2015), who measured happiness reactivity in FTD patients who watched a film clip designed to elicit happiness and by Perry et al. (2017), who administered a series of tasks involving pleasant, unpleasant, and neutral olfactory stimuli, to bvFTD patients. Sturm et al. (2015) showed that, contrary to the assumptions of the “valence hypothesis,” atrophy in predominantly left hemisphere fronto-striatal emotion regulation systems was associated with greater happiness facial behavior during the film designed to elicit happiness. Perry et al. (2017) showed (in agreement with the “right hemisphere hypothesis”), that the inability to subjectively differentiate the valence of pleasant and unpleasant odors, correlated with atrophy in the right ventral mid-insula and the right amygdala. Furthermore, (at variance with the “valence hypothesis”) they showed that positive rating of unpleasant odors correlated with left anterior insula and frontal pole atrophy.

## Discussion

The outcome of the present review strongly confirms results of previous investigations (e.g., Miller et al., 1993; Edwards-Lee et al., 1997; Seeley et al., 2005; Irish et al., 2013; Binney et al., 2016; Kumfor et al., 2016), which have shown that inappropriate social and emotional behaviors are an early and distinctive marker of the right-sided variants of FTLD. Furthermore, data reported in Table 2 corroborates our decision to consider emotional and behavioral disorders as distinct but highly interconnected phenomena, because a high prevalence of patients with right FTD were observed both in studies based on behavioral abnormalities, and in those evaluating specific aspects of emotional disorders. The former had been assessed clinically (evaluation of medical records) in papers 1. Miller et al. (1993), 2. Edwards-Lee et al. (1997), 3. Mychack et al. (2001), 8. Seeley et al. (2005) and 13. Chan et al. (2009) and with the Neuropsychiatric Inventory (Cummings et al., 1994) in studies 6. Liu et al. (2004), 7. Rosen et al. (2005) and 14. Massimo et al. (2009). The latter, on the contrary, had has often been investigated with specific testing procedures, such as the IRI measure of empathy (Davis, 1983) in studies 9. Rankin et al. (2006), 18. Eslinger et al. (2011) and 28. Dermody et al. (2016); the Awareness of Social Inference Test (TASIT / McDonald et al., 2002) in studies 15. Kipps et al. (2009), 16. Rankin et al. (2009), 19. Goodkind et al. (2012), 25. Downey et al. (2015) and 30. Multani et al. (2017); the TOM cartoons (Lough et al., 2006) in study 23. Irish et al. (2014); the Ekman 60-Faces task (Ek60F /Young et al., 2002) in study 24. Cerami et al. (2015); the Comprehensive Affect Testing System (CATS/ Froming et al., 2006) in study 27. Binney et al. (2016) and the Face and Emotion Processing Battery (Miller et al., 2012) in study 29. Kumfor et al. (2016). However, in spite of these important methodological differences between studies based on behavioral abnormalities, and those evaluating specific aspects of emotional disorders, a similarly high prevalence of right fronto-temporal lesions was found across these investigations. Some authors (e.g., Zahn et al., 2009; Ross and Olson, 2010; Olson et al., 2013) had interpreted these inappropriate social and emotional behaviors within the framework of the “Semantic Hub” model (Rogers et al., 2004; Patterson et al., 2007; Lambon Ralph and Patterson, 2008). However, in contrast with the original version of this model, that viewed the “Hub” as a bilaterally represented unitary mechanism, they had maintained that the left ATL supports general conceptual knowledge, whereas the right ATL subsumes a domain-specific social cognition system. This hypothesis was however questioned by results of a recent review in which Gainotti (2015) showed that the right ATL plays a more important role in behavioral and emotional functions than in higher level social cognition and considered these disorders as due to the disruption of lower level (behavioral and emotional) components than of higher level (social cognitive) aspects.

### Discussion of Investigations Relevant With Respect to the “Right Hemisphere Hypothesis”

Based on these premises, the present review aimed to evaluate if results of investigations conducted in patients with FTLD support one of the main models advanced to explain the relationship between emotions and brain laterality, namely the “right hemisphere hypothesis” or the “valence hypothesis.” The first model posits a general dominance of the right hemisphere for all kinds of emotions, while the second assumes an opposite dominance of the left hemisphere for positive emotions and of the right hemisphere for negative emotions. Results of the review clearly support the “right hemisphere hypothesis” more than the “valence hypothesis.” All the group comparisons that investigated the influence of asymmetrical atrophy on emotional and behavioral disorders in FTD patients demonstrated, in fact, a prevalence of these disorders in subjects with atrophy or hypometabolism of right-sided neural structures. Furthermore, results consistent with the “right hemisphere hypothesis” were obtained by all-but-one investigation (i.e., studies 9, 15, 16, 17, 18, 19, 21, 22, 23, 24, 25, 29, 30, and 32) that used a regression analyses to determine potential correlations between behavioral and emotional disturbances and lateralized impairment of specific brain structures. The high prevalence of behavioral abnormalities and of emotional disorders in patients with right FTLD, had been noted by previous authors, who tried to explore the reasons that could explain this association. Thus, Liu et al. (2004) suggested that the prevalence of behavioral disturbances in FTD patients with right-sided lesions may be related to the importance of the right hemisphere for the comprehension of emotional stimuli and an even more articulated interpretation of the links between poor comprehension of emotional facial expressions and behavioral abnormalities was offered by Lough et al. (2006) and Goodkind et al. (2015). These authors acknowledged that the inability of right FTD patients, to read the negative emotional expressions on the faces of personally relevant people, provoked by their socially inappropriate behavior, could contribute to the persistence of these abnormal social conducts. Thus, if a patient's behavior makes family members angry and this anger is correctly recognized, it might motivate the patient to modify his unacceptable conduct, but if this anger is not correctly recognized, improvement of the abnormal social behavior would be less likely. Obviously, if the reviewed data stress a general dominance of the right hemisphere for all kinds of emotions, they do not suggest that the left fronto-temporal structures play an irrelevant role in emotional functions. In fact, in one of the studies included in the present review (Dermody et al., 2016) the emotional defect (reduced empathic concern) in bvFTD patients was associated with prevalent atrophy in left-sided rather than right-sided structures (the left orbitofrontal and insular cortices). In another study (Couto et al., 2013) emotion deficits and Tome impairments correlated more with right- than with left-sided structures (insula and temporal pole) only in progressive non-fluent aphasia (PNFA), but not in bvFTD patients. Overall, in all the other correlational studies, emotional disorders were associated to atrophy of both the right- and left- anterior temporal, insular and orbitofrontal cortices, even though the correlations were stronger with right- than with left-sided structures. The relationship between emotional disorders and atrophy of right fronto-temporal structures is, on the other hand, confirmed by the fact that several studies included in our review (e.g., Rankin et al., 2006; Couto et al., 2013; Irish et al., 2014; Kumfor et al., 2016; Multani et al., 2017) have shown that, even in patients with bilateral or left lateralized forms of FTLD, the emotional aspects of interpersonal functioning correlate with atrophy in right rather than left temporal lobe structures.

### Discussion of Investigations Specifically Concerning the “Valence Hypothesis”

If results of investigations concerning the “right hemisphere hypothesis” had clearly supported that model, results of investigations concerning the “valence hypothesis” were, at best, inconclusive. As a matter of fact, only two of these investigations [namely the Rosen et al. (2006) and the Werner et al. (2007) studies] seemed to corroborate at least in part the “valence hypothesis,” because accuracy in recognition of negative emotions was positively correlated with tissue content of right temporal structures in the Rosen et al. (2006) study and with left frontal atrophy in the Werner et al. (2007) paper. In the Rosen et al. (2006) study, however, no lateralization was found with respect to positive emotions, which, according to the “valence hypothesis” should be subserved by left hemisphere structures. Similarly, in the Werner et al. (2007) paper the generation of both happy facial expressions to happy films and of sad facial expressions to sad films, were associated with greater lobar volume in the right frontal lobe (a finding more consistent with the “right hemisphere” than with the “valence hypothesis”). Furthermore, results in disagreement with the “valence hypothesis” were obtained by Rosen et al. (2002b), Irish et al. (2013), Sturm et al. (2015) and Perry et al. (2017). Rosen et al. (2002b) showed that the comprehension of facial expressions of happiness was significantly correlated with the right amygdala and the right orbito-frontal cortex (OFC) volume. Irish et al. (2013) proved that right SD patients scored significantly worse than left SD patients in the recognition of happy faces. Sturm et al. (2015) showed that the projection of a film clip designed to elicit happiness provoked greater happiness facial behavior in patients with left fronto-temporal atrophy and Perry et al. (2017) revealed that, in bvFTD patients, the tendency to give positive ratings of unpleasant odors correlated with atrophy of the left dorsal anterior insula and of the left frontal pole. On the basis of these results, and of data showing that happiness is the simplest facial emotional expression (Ekman, 2007), it is therefore possible to explain results obtained by Rosen et al. (2006) not only in terms of the “valence” but also of the “right hemisphere” hypothesis. In the Rosen et al. (2006) study, in fact, as in many similar investigations conducted on patients with degenerative brain diseases (e.g., Rosen et al., 2002b, 2004; Irish et al., 2013; Bora et al., 2016), performance on the recognition of facial expressions was poorer for negative emotions (fear, anger, and sadness) than for happiness. It is, therefore, possible that the correlation between right temporal structures and the recognition of negative emotions could be due to the greater right hemisphere involvement in the most difficult part of the task than to the (negative) valence of the emotions to be recognized. Rosen et al. (2006) have also noted that if participants recognize that a photograph portrays a negative emotion, they still have to figure out which of several negative emotions is being expressed. In contrast, if they recognize that a photograph is depicting a positive emotion, then it must be happiness. This explains the oft-reported findings that patients have more difficulty recognizing negative emotions than positive emotions. We can, therefore, conclude that results of the present review are more consistent with the “right hemisphere” than with the “valence hypothesis,” because the first model is supported by the outcome of all investigations considered as relevant with respect to the “right hemisphere” hypothesis and of most studies considered as relevant with respect to the “valence hypothesis.”

These conclusions are in accordance with both those of previous papers carried out on other neurological diseases (e.g., Gainotti, 1972; Coffey, 1987; Gainotti and Caltagirone, 1989; Borod et al., 1998; Mandal and Ambady, 2004) that have also supported the “right hemisphere hypothesis,” and with those of a recent paper, in which Gainotti (2018) has tried to rework the “right hemisphere hypothesis” in terms of brain-behavior relationships. As for previous papers, I will only mention here a review paper by Coffey (1987) and a research paper by Borod et al. (1998). Coffey (1987) surveyed clinical and experimental studies conducted with brain-damaged patients, normal subjects, and psychiatric patients and concluded that the right hemisphere is uniquely specialized for the perception, experience, and expression of emotion. Borod et al. (1998) examined emotional comprehension in right and left brain-damaged (BD) stroke patients and in normal controls across three communication channels (facial, prosodic, and lexical) and contrasted the “right-hemisphere” with the “valence hypothesis.” Right BD patients were significantly impaired relative to left BD patients and normal controls across channels and valences, supporting the RH hypothesis. As to the question of what these results could mean for brain-behavior relationships, Gainotti (2018) reviewed results of recent investigations which have ascertained a critical role: (a) of the right amygdala in the unconscious processing of emotional information; (b) of the right ventromedial prefrontal cortex in functions of emotional control, and (c) of the right anterior insula in the conscious experience of emotions. All these data therefore support and provide an updated, congruent version of early models assuming a general dominance of the right hemisphere for emotional functions.

### Limitations of the Study

The main limitation of this review probably consists in the heterogeneity of studies included in our survey, because the sources of evidence utilized were different with respect to the content and the methodology used in different studies. As for content related differences, they concerned the different syndromes (e.g., fv FTD vs. tvFTD or bvFTD vs. SD) belonging to the spectrum of FTLD diseases considered in different studies and the (behavioral or properly emotional) nature of the disorders investigated. With respect to the different syndromes considered, it must be noted that the location of disease may be variable in different patients, because lesions are generally bilateral, and there is varying involvement of the frontal and temporal lobes (Kumfor et al., 2016). As for the nature of the disorders investigated, even within studies dealing with true emotional disorders, two main sources of heterogeneity can be underlined. The first refers to the fact that some studies investigated basic emotions, considered as innate and with high survival values (Ekman, 1984, 1992) and in other cases “complex,” “social,” and “self-conscious emotions” subtended by social norms (Ekman, 1984, 1992) and requiring an appreciation of the self in a social context (Tangney, 1999). The second source of heterogeneity refers to the fact that some studies made a global assessment of emotional disorders, whereas other studies made a distinction between negative and positive emotions. With respect to the methodology, the review included: (a) studies based on the comparison between left-sided and right-sided atrophy in FTD patients, with emotional or behavioral disorders and (b) studies based on the assessment of the correlations between specific emotional disorders and atrophy or hypometabolism of equally specific left-sided or right-sided structures. Even if these sources of heterogeneity did not allow for a meta-analysis of data gathered in patients with FTD or other degenerative brain diseases, results of the present review can be considered as substantial proof to support (at the present state of our knowledge) the “right hemisphere” rather than the “valence hypothesis.”

## Author Contributions

The author confirms being the sole contributor of this work and has approved it for publication.

### Conflict of Interest Statement

The author declares that the research was conducted in the absence of any commercial or financial relationships that could be construed as a potential conflict of interest.
